# Supported bridge position in one‐stop coronary and craniocervical CT angiography: A randomized clinical trial

**DOI:** 10.1002/acm2.14561

**Published:** 2024-11-15

**Authors:** Heng Zhou, Cheng Yan, Min Ji, Zhang Shi, Chun Yang, Mengsu Zeng

**Affiliations:** ^1^ School of Health Science and Engineering University of Shanghai for Science and Technology Shanghai China; ^2^ Department of Radiology Zhongshan Hospital Fudan University Shanghai China; ^3^ Shanghai Institute of Medical Imaging Shanghai China; ^4^ Shanghai United Imaging Healthcare Co., Ltd. Shanghai China

**Keywords:** artery, carotid artery disease, cerebral artery disease, computed tomography angiography, coronary artery disease, patient arm position

## Abstract

**Objective:**

The routine patient arm position differs between coronary CT angiography (CTA) and craniocervical CTA protocols. To investigate the clinical feasibility of supported bridge position (SBP) in combined coronary and craniocervical CTA.

**Methods:**

Prospective enrollment included patients with suspected coronary artery disease (CAD) or craniocervical artery disease (CCAD) from February 2022 to November 2022. Patients were divided into three groups: coronary or craniocervical CTA according to CAD or CCAD using standard position (group 1), combined CTA with naturally arm‐down position (group 2) and SBP (group 3). Statistical analysis of objective image quality, such as noise and contrast‐to‐noise ratio (CNR), subjective image quality, patient position and radiation dose was performed among the three groups.

**Results:**

Two hundred and one patients (median age, 67 years; 138 men) were included. In terms of CNR for cardiac segment, group 1 and group 3 had no statistical difference, both significantly higher than group 2 (group 1, 12.56 ± 2.05; group 2, 10.4 ± 2.43; group 3, 11.94 ± 2.22; *P* < 0.05). Subjective image evaluation revealed no statistically significant differences among the three groups of coronary arteries (*P* > 0.05). Additionally, the lateral project value of scout images at the heart level indicated a significant difference (119.48 ± 12.19, 182.34 ± 25.09, and 140.58 ± 19.68 of patients, for group 1, group 2, and group 3, respectively, *P* < 0.05). No statistical differences were observed in ${\mathrm{CTDI}}_{\mathrm{vol}}$ between group 1 and group 3 (cardiac scan, 15.77 [15.07–16.37] mGy vs. 14.88 [12.19–18.81] mGy; craniocervical scan, 7.85 [7.69–8.01] mGy vs. 7.88 [7.88–7.89] mGy; all *P* > 0.05). However, group 2 had a higher dose (19.54 [16.86–22.85] mGy and 10.87 [10.86–10.87] mGy, for cardiac and craniocervical scans, respectively).

**Conclusions:**

In comparison with a naturally arm‐down position, SBP, which aligns the humerus bones with the spinal column, can provide diagnostic image quality at routine dose level of standard position CTA.

## 1 INTRODUCTION

Coronary heart disease and stroke are the top causes of morbidity and mortality worldwide.^1^, ^2^ Various connections between craniocervical artery disease (CCAD) and coronary artery disease (CAD) have been found. Previous research has highlighted the significant correlation between the coronary plaque phenotype and the composition of carotid plaques.^3^ Furthermore, the severity of craniocervical artery stenosis and the extent of craniocervical atherosclerosis were strongly associated with 50% asymptomatic CAD.^4^ CAD emerges as a significant contributor to mortality among patients with cerebrovascular disease.^5^ Several studies have shown that: patients with a history of ischemic stroke often present with asymptomatic CAD; one‐third of ischemic stroke patients without cardiac disease history exhibiting coronary stenosis exceeding 50%^6^; presymptomatic cardiac disease is prevalent in a substantial portion of patients with stroke or transient ischemic attack, ranging from 20% to 40%^7^, ^8^; cardiac adverse events are common and begin to occur shortly after the onset of stroke.^9^ Hence, identifying occult CAD in patients with CCAD holds considerable clinical importance.

Coronary computed tomography angiography (CTA) is a widely used and effective technique for evaluating CAD.^10^, ^11^ The limitations of previous CT scanners constrained research on the combined CTA protocol.^12^ The advent of dual‐source CT technology improved temporal resolution and dose efficiency.^13^, ^14^, ^15^ The subsequent introduction of wide‐detector CT systems enabled scanning of the entire heart within one heartbeat.^16^ All these technological advancements have optimized coronary CTA and prompt further research on the combined coronary and craniocervical CTA.^17^, ^18^

Earlier studies have predominantly focused on the feasibility of combined coronary and craniocervical CTA, the positioning of the arms was not well discussed and either image quality or radiation exposure was compromised.^12^, ^19^ Because high attenuation of the arms will induce extra image noise and streaking artifacts, patients place their arms above their heads during routine coronary CTA,^20^ whereas for craniocervical CTA patients naturally place their arms at their sides. Thus, the optimal patient position was not clear for the clinical application of combined coronary and craniocervical CTA.

**FIGURE 1:**
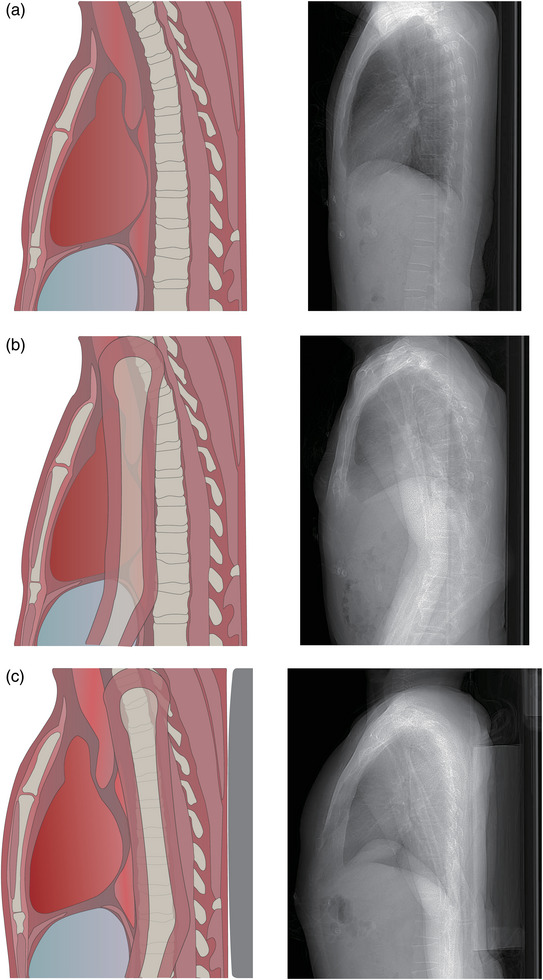
Illustration of three patient position during coronary scanning (left: schematic diagram; right: sample scout image), the standard coronary CTA position (group 1) (a), natural arm down position (group 2) (b), and the supported bridge position (group 3) (c).

For the further application of combined scanning in clinical practice, we proposed a supported bridge position (SBP, SBP is formed as follows: the arms are placed at the sides, but a support board is positioned under the patient's back to form a forced arching back [Figure 1]) in the combined coronary and craniocervical CTA in this study. The primary objective of this study was to evaluate the image quality and radiation dose of combined coronary and craniocervical CTA with SBP.

## 2 MATERIAL AND METHODS

### 2.1 Study population

This prospective study was approved by the Ethics Committee of Hospital, and written informed consent was obtained from all participants. Between February 2022 and November 2022, 212 consecutive patients with known or suspected CAD or CCAD were enrolled in the study. Patients with a history of severe allergic reactions to iodinated contrast, impaired renal function, or pregnancy were excluded from the study.

The enrolled patients were finally randomized and divided into three groups: standard coronary CTA (51 patients) and standard craniocervical CTA (50 patients) as group 1, combined coronary and craniocervical CTA (group 2, 52 patients), combined coronary and craniocervical CTA with SBP (group 3, 48 patients).

### 2.2 Scan protocol

The examinations were conducted using a 320‐row detector CT scanner (solid‐state ceramic scintillator detector, uCT960+, United Imaging Healthcare). The combined CTA protocol started with coronary CTA. After completing the coronary CTA and 2.5‐s switching time, a craniocervical CTA was performed. Administer 25–50 mg of metoprolol tartrate 1–2 h prior to the examination (if heart rate > 65 beats/min), followed by 0.5 mg of sublingual nitroglycerin 5 min before the examination.

Coronary CTA acquisition parameters were as follows: 320 × 0.5 mm collimation, 0.25 s rotation time, axial scan, 100 kVp, the tube current was recommended by the adaptive system and manually determined the mA based on patient BMI, and the scanning range started from the tracheal bifurcation to the diaphragm below all cardiac structures. The R‐R interval range was automatically selected by the system according to the patient's heart rate. The acquisition parameters of craniocervical CTA were as follows: 160 × 0.5 mm collimation, 0.25 s rotation time, helical scan (pitch = 0.8938), 100 kVp, the tube current was recommended by the adaptive system, and the scanning range started from the aortic arch to the vertex of the head. Furthermore, the scanning protocols in three groups involve both coronal and sagittal scout scans, covering the region from head to abdomen. The only variation in scanning parameters among the three groups was the tube voltage. Specifically, both for coronary and craniocervical CTA, group 1 and group 3 were 100 kVp, whereas 120 kVp was for group 2.

Patients received an iomeprol injection (350 mg iodine/mL; from Shanghai Borui KeXinYi Pharmaceutical Co) through a high‐pressure injector in the cubital vein, followed by the infusion of 0.9% sodium chloride solution. The standard coronary CTA was initiated 5 s after the contrast reached 120 HU in the descending aorta. The standard craniocervical CTA was initiated 4.5 s after the contrast reached 120 HU in the descending aorta. For groups 2 and 3, the combined protocol was initiated 5 s after the contrast reached 120 HU in the descending aorta. The injection rates and total contrast agent volumes varied across groups: 4 mL/s for coronary or craniocervical CTA in group 1, 5 mL/s for group 2, and 4 mL/s for group 3. The volumes were calculated based on body weight: 0.6 * body weight (in kg) for coronary or craniocervical CTA in group 1, 0.8 * body weight (in kg) for group 2, and 0.75 * body weight (in kg) for group 3. A total volume of 25–30 mL was used for the sodium chloride solution in each case. The bolus‐tracking technique with a region of interest (ROI) was utilized during the examinations to monitor the injection.

### 2.3 Patient positioning

All scans were performed in a supine position with the feet first in. In group 1, patients underwent coronary CTA with arms crossed above their heads, or craniocervical CTA with arms naturally resting at their sides. Patients in group 2 performed combined CTA with arms naturally at the sides. Patients in group 3 also performed combined CTA, but with SBP.

### 2.4 Image reconstruction

The coronary CTA images were reconstructed with the following parameters: 200 mm field of view (FoV), 512 × 512 matrix, a slice thickness and interval of 0.5 mm, C_SOFT_AA kernel, and level 8 of hybrid iterative reconstruction algorithm (KARL3D, United Imaging Healthcare). The software automatically determined the best reconstruction phase for coronary CTA reconstruction (ePhase, United Imaging Healthcare). The craniocervical CTA images were reconstructed with the following parameters: 230 mm field of view (FoV), 512 × 512 matrix, a slice thickness and interval of 0.5 mm, H_SOFT_B kernel, and level 8 of hybrid iterative reconstruction algorithm (KARL3D, United Imaging Healthcare).

### 2.5 Quantitative analysis

The measurements were performed three times by a radiologist (Y.C; with more than 6 years of experience in cardiovascular and cerebrovascular imaging) on the workstation (uOmnispace.MI, R001, United Imaging Healthcare, Shanghai, China) and then averaged to reduce measuring inaccuracies. The observer was blinded to the scanning protocol. For the objective evaluation of coronary arteries, the ${\mathrm{HU}}_{\mathrm{artery}}$ value was measured by placing ROI in the following places: left main, left anterior descending, left circumflex, right coronary artery, distal right coronary artery, ascending aorta, and descending aorta. To define vessel contrast, ${\mathrm{HU}}_{\mathrm{tissue}}$ value was measured within ROI placed in the surrounding soft tissue (size, 25 ${\mathrm{mm}}^{2}$). Image noise was defined as the standard deviation within ROI at the left atrium (size, 300 ${\mathrm{mm}}^{2}$). For the craniocervical arteries, the ROIs were positioned at the segments: the shoulder level—aortic arch and pectoralis major; the base of neck level—an original segment of the common carotid artery, preforaminal segment of the vertebral artery and sternocleidomastoid muscle; the neck level—common carotid artery at the bifurcation, foraminal segment of the vertebral artery and sternocleidomastoid muscle; the brainstem level—internal carotid artery, basal artery, and parenchyma; and the cerebral level—M1 segment of the middle cerebral artery and parenchyma.^19^, ^21^ The following formulas were used to quantify image quality: signal‐to‐noise ratio (SNR) = ${\mathrm{HU}}_{\mathrm{artery}}$/noise and contrast‐to‐noise ratio (CNR) = (${\mathrm{HU}}_{\mathrm{artery}}$—${\mathrm{HU}}_{\mathrm{tissue}}$)/noise. The ROI size was configured to approximately 1/2 to 2/3 of the artery diameter. ROI were positioned to avoid vessel edges, stenosis, or calcification plaques. In the case of smaller vessels, the ROIs were set to a minimum diameter of 2 mm to ensure accurate measurements. Figure S1 illustrates the measurement process.

By definition, a scout image is an x‐ray projection image and the pixel value is defined as μl−1000 in which μ is effective attenuation coefficient and *l* is penetration thickness. Thus, to estimate the lateral and anterior‐posterior thickness of patients, the pixel value or projection value (PV) of scout images was measured. We outlined ROIs at the maximum diameter of the heart in both coronal and sagittal views, recording their average values + 1000 as PV.

### 2.6 Qualitative analysis

All images were analyzed on the workstation (uOmnispace.MI, R001, United Imaging Healthcare, Shanghai, China). The assessment of coronary arteries involved dividing the coronary artery into 16 segments.^22^ The right coronary artery comprised segments 1 to 4, the left main was designated as segment 5, the left anterior descending included segments 6 to 10, and the left circumflex encompassed segments 11 to 15. Additionally, the ramus intermedius, if present, was identified as segment 16.^23^ To assess the craniocervical arteries, the following arterial segments were examined: the common carotid artery, the foraminal segments of the vertebral artery, the internal carotid artery, the basal artery, and the M1 segment of the middle cerebral artery.

Two radiologists (S.Z. and Y.C.), both with more than 5 years of experience in cardiovascular and cerebrovascular imaging, independently analyzed the CTA images in a randomized order. Observers were kept unaware of the scanning protocol. Image evaluation utilized axial images and curved multiplanar reformats. An image quality score by a 4‐point scale, as follows: Score 1, nondiagnostic: severe artifacts, insufficient contrast, or unclear vascular delineation; Score 2, adequate: evident artifacts with blurred vascular contours; Score 3 good: minor artifacts with relatively clear vascular contours; Score 4 excellent: absence of motion artifacts, significant vascular attenuation and clear contours of the vessels.

### 2.7 Radiation dose assessment

The radiation dose parameters, including ${\mathrm{CTDI}}_{\mathrm{vol}}$ and DLP, were automatically documented upon completion of each CT scan. The effective dose of CTA was determined by multiplying the DLP by the conversion coefficient (chest: *k* = 0.014 $\mathrm{mSv}\times {\left[\mathrm{mGy}\times \mathrm{cm}\right]}^{-1}$; craniocervical: *k* = 0.0031 $\mathrm{mSv}\times {\left[\mathrm{mGy}\times \mathrm{cm}\right]}^{-1}$).^24^, ^25^

### 2.8 Statistical analysis

Statistical analysis was conducted utilizing SPSS (version 25). Categorical variables were depicted as frequencies and percentages. To assess the normal distribution of continuous variables, the Shapiro–Wilk test was employed. Normally distributed data were represented as means ± standard deviation, while non‐normally distributed variables were displayed as medians and interquartile ranges. Pearson's chi‐squared test was utilized to analyze categorical data proportions. For continuous normally distributed variables, one‐way ANOVA was employed, whereas the Kruskal–Wallis H Test was utilized for nonparametric variables. Inter‐rater consistency was evaluated using the kappa test. A *P*‐value ≤ 0.05 was considered statistically significant.

## 3 RESULTS

### 3.1 Patient characteristics

**FIGURE 2:**
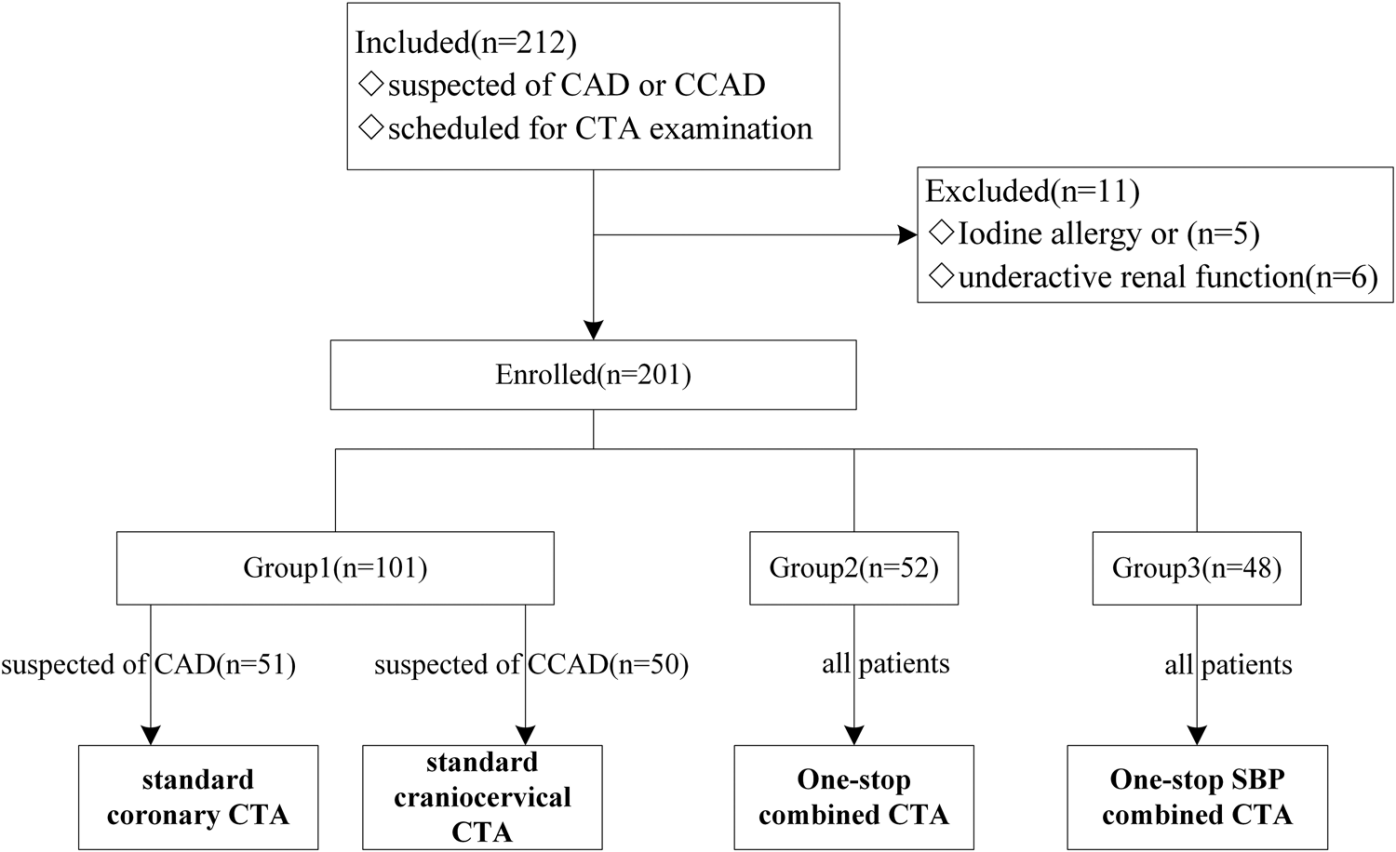
Flowchart for the patient enrollment and study design. SBP, supported bridge position.

Initially, 212 patients were considered for the study, but only 201 patients were ultimately enrolled in the investigation cohort, as shown in Figure 2. Detailed patient characteristics are provided in Table 1. There was no significant difference between patients for the three groups with respect to age, the male/female ratio, height, weight, or BMI. The heart rate in group 3 was comparable with group 1 (*P* = 0.36) and significantly higher than group 2 (*P* = 0.02).

**TABLE 1:** Patient characteristics.

	Group 1				
Characteristic	Craniocervical	Coronary	Group 2	Group 3	*P*
Age (years)	69.00 (61.00–73.00)	65.00 (57.00–69.00)	67.00 (56.00–73.75)	67.50 (56.25–75.00)	0.25
Gender (male/female)	34/16	35/16	38/14	31/17	0.84
Hight (cm)	166.59 ± 5.07	167.35 ± 9.37	167.93 ± 6.70	165.65 ± 7.19	0.38
Weight (kg)	65.59 ± 9.24	68.83 ± 13.29	66.83 ± 8.60	66.10 ± 9.82	0.72
BMI ($\mathrm{kg}/m^{2}$)	23.68 ± 3.57	24.51 ± 3.91	23.73 ± 3.08	24.06 ± 3.14	0.6
Heart rate (bpm)	NA	66.00 (60.00–77.00)	62.50 (58.00–73.00)	71.00 (63.25–85.50)	0.01

### 3.2 Quantitative image quality analysis

**FIGURE 3:**
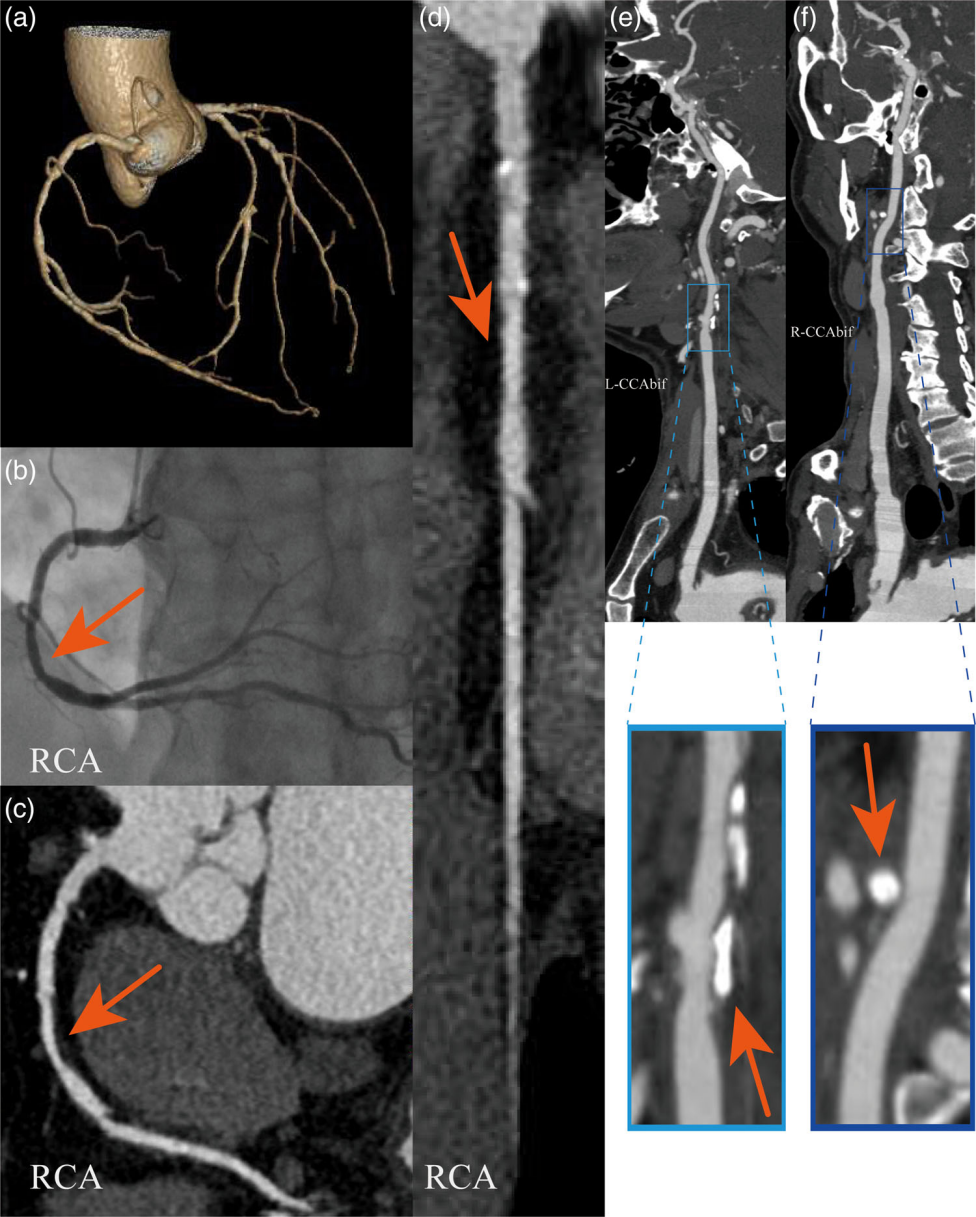
Images of VR (a), DSA (b), coronary CTA (c, d), and craniocervical CTA (e, f), for a patient in performed combined coronary and craniocervical CTA. A 72‐year‐old male presented with chest tightness. The coronary CTA (c‐d) displays both soft and calcified plaques in the RCA, with a moderate luminal narrowing. The coronary DSA (b) illustrates moderate narrowing in the RCA, consistent with the findings in coronary CTA. Craniocervical CTA (e, f) presents calcified plaques at the $R-{\mathrm{CCA}}_{\mathrm{bif}}$ and $L-{\mathrm{CCA}}_{\mathrm{bif}}$. ${\mathrm{CCA}}_{\mathrm{bif}}$, the common carotid artery at the bifurcation; DSA, digital subtraction angiography; RCA, right coronary artery; VR, volume rendering.

**FIGURE 4:**
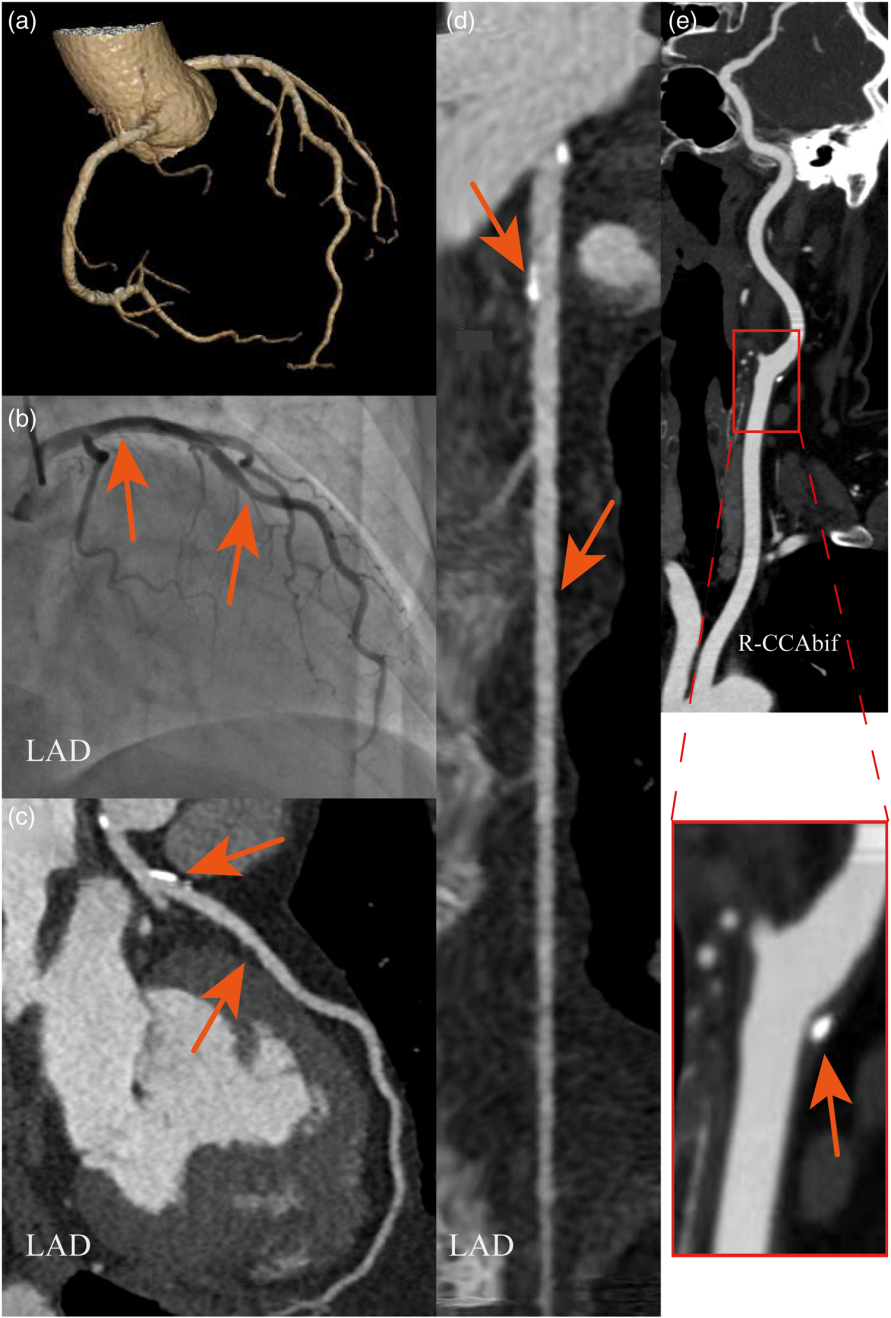
Images of VR (a), DSA (b), coronary CTA (c, d), and craniocervical CTA (e), for a patient performed combined coronary and craniocervical CTA with supported bridge position. A 64‐year‐old woman presented with symptoms of chest tightness and pain. The coronary CTA (c, d) reveals calcified plaques in the LM and LAD, with slight luminal narrowing. The coronary DSA (b) exhibits plaques in the LM and mild narrowing in the LAD, consistent with the findings in coronary CTA. Craniocervical CTA (e) shows calcified plaques at the $R-{\mathrm{CCA}}_{\mathrm{bif}}$. ${\mathrm{CCA}}_{\mathrm{bif}}$, the common carotid artery at the bifurcation; DSA, digital subtraction angiography; LAD, left anterior descending; LM, left main; VR, volume rendering.

All CTA images were diagnostically acceptable, demonstrating no significant artifacts or inadequate delineation of the lumen and surrounding tissues. Representative images obtained using combined CTA or SBP combined CTA are shown in Figures 3 and 4.

**FIGURE 5:**
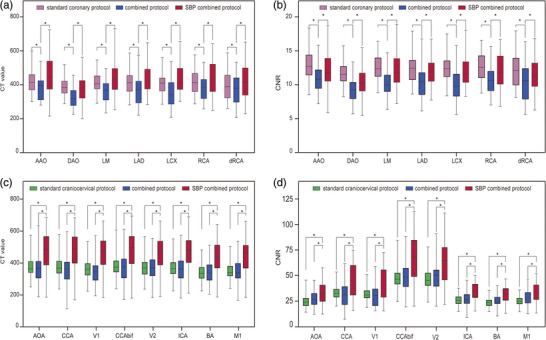
Objective image quality comparison among the three groups. (a) and (b) respectively show the CT values and CNR of the cardiac regions across the three groups; (c) and (d) respectively show the CT values and CNR of the craniocervical segments across the three groups. Asterisk (*) denotes statistical significance (*P* < 0.05). AAO, ascending aorta; AOA, ascending aorta; BA, basal artery; CCA, original segment of the common carotid artery; ${\mathrm{CCA}}_{\mathrm{bif}}$, the common carotid artery at the bifurcation; DAO, descending aorta; dRCA, distal right coronary artery; ICA, internal carotid artery; LAD, left anterior descending; LCX, left circumflex; LM, left main; M1, the M1 segment of the middle cerebral artery; RCA, right coronary artery; SBP, supported bridge position; V1, preforaminal segment of the vertebral artery; V2, foraminal segment of the vertebral artery.

Comparisons of the objective image quality among the three groups are depicted in Table 2 and Table S1. For the CT values of coronary arteries, group 3 showed similarity to group 1, with no statistical difference (all *P* > 0.05). However, group 2 yielded significantly lower values than group 1 and group 3 (all *P* < 0.05). Regarding the image noise in the left atrium, group 2 and group 3 exhibited similarity and without a statistical difference (32.28 (31.11–34.4) versus 33.5 (32.11–35.95), *P* = 0.22). In terms of CNR for coronary arteries, group 1 and group 3 demonstrated no statistical difference (all *P* > 0.05), while group 2 exhibited significantly lower values compared to the other two groups (all *P* < 0.05) (Figure 5).

The CT values, SNR, and CNR of various segments in the craniocervical arteries showed similarity between group 1 and group 2, with no statistical difference (all *P* > 0.05). However, group 3 exhibited higher values compared to group 1 and group 2, and this difference was statistically significant (all *P* < 0.05).

### 3.3 Qualitative image quality

**FIGURE 6:**
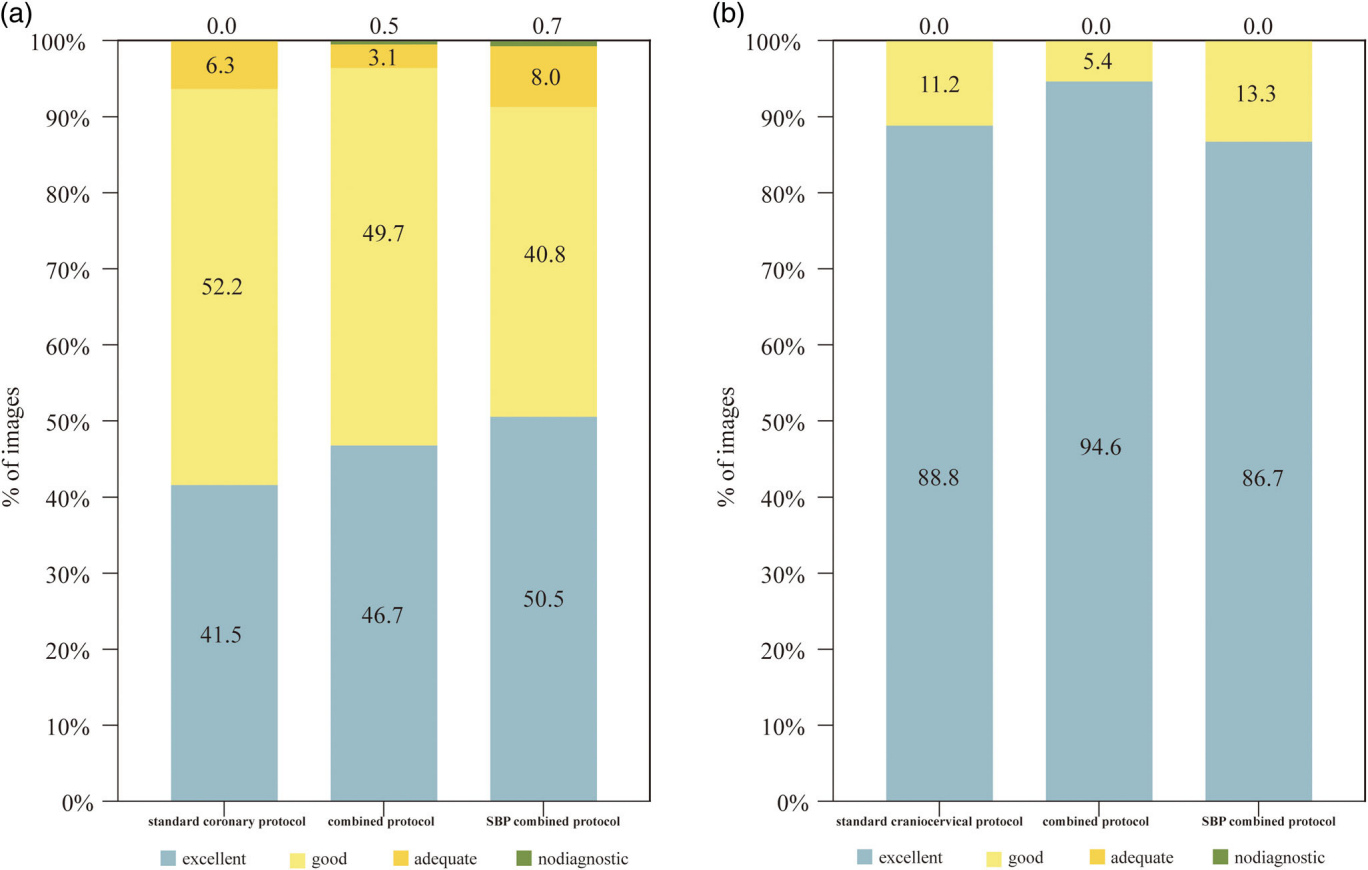
Subjective image quality assessment results for coronary arteries (a) and craniocervical arteries (b) in three groups. SBP, supported bridge position.

A total of 2189 coronary artery segments and 750 craniocervical artery segments, each with a vessel diameter of at least 1.5 mm, were available for evaluation. Excellent inter‐reader agreement (*k* = 0.861) was observed in the subjective image quality assessment of craniocervical and coronary arteries. The comprehensive results of the qualitative image quality assessments for both coronary and craniocervical segments are presented in Table S2. Subjective image evaluation revealed no statistically significant differences among the three groups of coronary arteries (3.29 ± 0.49, 3.36 ± 0.53, and 3.31 ± 0.58, for group 1, group 2, and group 3, respectively, *P* > 0.05). Similarly, the craniocervical arteries of three groups exhibited comparable subjective image evaluation (group 1, 3.92 ± 0.24; group 2, 3.96 ± 0.19; group 3, 3.9 ± 0.26; *P* > 0.05) (Figure 6).

### 3.4 Patient positioning

The PV in coronal position was not significantly different among the three groups (134.15, 138.55, and 136.78 of patients, for group 1, group 2, and group 3, respectively, *P* > 0.05). For the PV in sagittal position, significant differences were found among the three groups (group 1, 119.48 ± 12.19; group 2, 182.34 ± 25.09; group 3, 140.58 ± 19.68; *P* < 0.05) (Table 3). The variation between the image noise of the left atrium and the image noise of the ascending aorta showed no statistical difference in group 1 and group 3 (1.42 (0.95–2.48) vs. 1.2 (0.7–1.75), *P* > 0.99), and notably lower than those in group 2. A linear regression equation was developed, incorporating PV in a sagittal position (Figure S2). The results indicated that the image noise of the left atrium was proportional to the PV in the sagittal position (*b* = 0.018, *t* = 2.9, *P* = 0.004).

### 3.5 Radiation dose

The CT scan parameters are summarized in Table 4. For both coronary and craniocervical regions, ${\mathrm{CTDI}}_{\mathrm{vol}}$, DLP and effective dose exhibited similarity between group 1 and group 3, with no statistical difference (all *P* > 0.05), and both were significantly lower than group 2 (all *P* < 0.05).

**TABLE 2:** Quantitative image quality analysis.

		Group 1	Group 2	Group 3	*P*‐value	$P_{1}$	$P_{2}$	$P_{3}$
HU	AAO	438.79 ± 62.87	398.86 ± 61.94	462.63 ± 100.75	0.002	0.04	>0.99	0.002
	DAO	406.23 ± 51.64	346.07 ± 54.33	393.68 ± 76.86	<0.001	<0.001	>0.99	0.001
	LM	432.48 ± 56.18	380.44 ± 62.24	458.87 ± 93.02	<0.001	0.001	0.94	<0.001
	LAD	430.86 ± 63.54	385.67 ± 80.78	445.74 ± 75.82	<0.001	0.002	0.32	<0.001
	LCX	426.13 ± 56.92	370.50 ± 75.41	447.18 ± 78.34	<0.001	<0.001	0.14	<0.001
	RCA	432.96 ± 64.35	396.41 ± 62.75	454.16 ± 95.34	0.004	0.05	>0.99	0.004
	dRCA	420.30 ± 80.76	389.24 ± 81.66	435.65 ± 94.69	0.02	0.07	0.38	0.008
	AOA	377.26 ± 58.77	364.14 ± 80.64	479.98 ± 117.88	<0.001	>0.99	<0.001	<0.001
	CCA	379.12 ± 61.19	354.07 ± 81.32	482.12 ± 122.09	<0.001	0.86	<0.001	<0.001
	V1	365.07 ± 57.84	342.74 ± 73.17	459.46 ± 113.63	<0.001	0.54	<0.001	<0.001
	${\mathrm{CCA}}_{\mathrm{bif}}$	383.04 ± 62.74	369.93 ± 81.10	492.50 ± 122.33	<0.001	>0.99	<0.001	<0.001
	V2	374.53 ± 59.75	368.95 ± 73.89	469.81 ± 114.47	<0.001	>0.99	<0.001	<0.001
	ICA	368.31 ± 59.48	356.84 ± 76.16	473.49 ± 109.67	<0.001	>0.99	<0.001	<0.001
	BA	340.45 ± 57.23	339.09 ± 61.79	433.58 ± 104.89	<0.001	>0.99	<0.001	<0.001
	M1	351.67 ± 54.05	344.72 ± 70.36	448.84 ± 110.65	<0.001	>0.99	<0.001	<0.001
CNR	AAO	12.85 ± 2.08	10.82 ± 1.98	12.31 ± 2.66	<0.001	<0.001	0.24	0.001
	DAO	11.77 ± 1.71	9.22 ± 1.74	10.32 ± 2.14	<0.001	<0.001	<0.001	0.004
	LM	12.64 ± 1.91	10.27 ± 2.04	12.22 ± 2.46	<0.001	<0.001	0.33	<0.001
	LAD	12.58 ± 2.13	10.44 ± 2.62	11.86 ± 2.05	<0.001	<0.001	0.12	0.002
	LCX	12.43 ± 1.93	9.99 ± 2.50	11.89 ± 2.08	<0.001	<0.001	0.22	<0.001
	RCA	12.65 ± 2.11	10.77 ± 2.12	12.07 ± 2.54	<0.001	<0.001	0.2	0.005
	dRCA	12.24 ± 2.70	10.54 ± 2.62	11.54 ± 2.60	0.006	0.001	0.19	0.06
	AOA	24.05 (20.45–27.71)	26.49 (21.63–32.40)	32.19 (24.69–40.65)	<0.001	0.33	<0.001	0.02
	CCA	33.34 ± 6.48	31.40 ± 11.81	45.83 ± 17.32	<0.001	0.86	<0.001	0.003
	V1	30.99 (28.05–35.16)	30.25 (20.47–36.84)	40.10 (28.60–55.75)	<0.001	0.88	<0.001	0.005
	${\mathrm{CCA}}_{\mathrm{bif}}$	46.53 (41.64–52.30)	50.00 (39.11–58.92)	68.21 (48.97–84.66)	<0.001	>0.99	<0.001	<0.001
	V2	45.31 (40.22–52.43)	50.64 (39.50–60.39)	64.86 (45.49–77.77)	<0.001	0.89	<0.001	0.001
	ICA	25.70 ± 4.88	26.90 ± 6.73	34.86 ± 8.55	<0.001	0.97	<0.001	<0.001
	BA	23.51 ± 4.72	25.38 ± 5.62	31.58 ± 7.97	<0.001	0.32	<0.001	<0.001
	M1	25.13 ± 4.58	28.02 ± 7.36	33.02 ± 8.84	<0.001	0.08	<0.001	0.02

*Notes*: The significance between group 1 and group 2 is represented by $P_{1}$, the significance between group 1 and group 3 is represented by $P_{2}$, and the significance between group 2 and group 3 is represented by $P_{3}$. All pairwise comparisons underwent Bonferroni correction.

Abbreviations: AAO, ascending aorta; AOA, ascending aorta; BA, basal artery; CCA, original segment of the common carotid artery; ${\mathrm{CCA}}_{\mathrm{bif}}$, the common carotid artery at the bifurcation; DAO, descending aorta; dRCA, distal right coronary artery; ICA, internal carotid artery; LAD, left anterior descending; LCX, left circumflex; LM, left main; M1, the M1 segment of the middle cerebral artery; RCA, right coronary artery; V1, preforaminal segment of the vertebral artery; V2, foraminal segment of the vertebral artery.

**TABLE 3:** Patients positioning and image noise.

	Group 1	Group 2	Group 3	*P*	$P_{1}$	$P_{2}$	$P_{3}$
PV‐CP	134.15 (125.35–144.10)	138.55 (128.09–143.99)	136.78 (129.75–144.55)	0.68	NA	NA	NA
PV‐SP	119.48 ± 12.19	182.34 ± 25.09	140.58 ± 19.68	<0.001	<0.001	<0.001	<0.001
AAO‐IN	29.20 ± 1.65	28.58 ± 1.91	32.47 ± 2.65	<0.001	0.67	<0.001	<0.001
LA‐IN	30.15 (29.65–30.95)	32.28 (31.11–34.40)	33.50 (32.11–35.95)	<0.001	<0.001	<0.001	0.22
IC	1.20 (0.70–1.75)	4.32 (3.07–5.66)	1.42 (0.95–2.48)	<0.001	<0.001	>0.99	<0.001

*Notes*: The significance between group 1 and group 2 is represented by $P_{1}$, the significance between group 1 and group 3 is represented by $P_{2}$, and the significance between group 2 and group 3 is represented by $P_{3}$. All pairwise comparisons underwent Bonferroni correction.

Abbreviations: AAO‐IN, image noise in the ascending aorta; LA‐IN, image noise in the left atrium; PV‐CP, project value in coronal position; PV‐SP, project value in sagittal position; IC, image consistency, difference between LA‐IN and AAO‐IN.

**TABLE 4:** Scan parameters.

	Parameter	Group 1	Group 2	Group 3	*P*	$P_{1}$	$P_{2}$	$P_{3}$
Coronary	${\mathrm{CTDI}}_{\mathrm{vol}}$ (mGy)	15.77 (15.07–16.37)	19.54 (16.86–22.85)	14.88 (12.19–18.81)	<0.001	<0.001	>0.99	<0.001
	DLP (mGy.cm)	248.15 (230.71–260.42)	312.64 (269.72–365.56)	238.12 (195.03–300.89)	<0.001	<0.001	>0.99	<0.001
	ED (mSv)	3.47 (3.23–3.65)	4.38 (3.78–5.12)	3.33 (2.73–4.21)	<0.001	<0.001	>0.99	<0.001
Craniocervical	${\mathrm{CTDI}}_{\mathrm{vol}}$ (mGy)	7.85 (7.69–8.01)	10.87 (10.86–10.87)	7.88 (7.88–7.89)	<0.001	<0.001	>.099	<0.001
	DLP (mGy.cm)	382.45 (361.79–399.31)	544.90 (528.16–577.82)	402.70 (382.57–414.85)	<0.001	<0.001	0.07	<0.001
	ED (mSv)	1.19 (1.12–1.24)	1.69 (1.64–1.79)	1.24 (1.18–1.29)	<0.001	<0.001	0.07	<0.001

*Notes*: The significance between group 1 and group 2 is represented by $P_{1}$, the significance between group 1 and group 3 is represented by $P_{2}$, and the significance between group 2 and group 3 is represented by $P_{3}$. All pairwise comparisons underwent Bonferroni correction.

$\mathrm{Abbreviations}:\;{\mathrm{CTDI}}_{\mathrm{vol}}$, volume computed tomography dose index; DLP, dose‐length product; ED, effective dose.

## 4 DISCUSSION

In this prospective study, we assessed the image quality and radiation dose associated with SBP in combined CTA of coronary and craniocervical arteries. We found that in comparison with the naturally arm‐down position, SBP could provide diagnostic image quality for coronary arteries (CNR: standard position, 12.56 ± 2.05; arm‐down, 10.4 ± 2.43; SBP, 11.94 ± 2.22; *P* < 0.05) by avoiding image noise and streaking artifacts at regular coronary CTA dose level.

Although the existence of research on combined coronary and craniocervical CTA, these studies lacked detailed descriptions of arm positions and neglected its impact on the image quality.^19^ Previous studies have explored the effect of patient position during CT scanning, suggesting that optimal patient position enabled diagnostic image quality with lower radiation dose.^26^, ^27^ In one research of the combined coronary and craniocervical CTA, patients' arms were positioned above the head to ensure cardiac image quality.^13^ However, this method resulted in substantial image noise and streaking artifacts in the craniocervical segment. Given the complex structures and numerous blood vessels, a craniocervical segment may require higher image quality compared with the cardiac region, thus its application requires further evaluation. On the another hand, if the patient's arms are naturally placed at their sides, excessive noise and streaking artifacts will be induced in the cardiac section and the application of 120 kVp is required to mitigate the problems. Meanwhile, to ensure the consistency of contrast enhancement, 120 kVp was also required for the craniocervical region when using 120 kVp for the cardiac region in the combined protocol. This will result in a higher contrast agent injection rate, contrast agent volumes, and radiation dose. However, our study introduces a novel approach to improve the arm‐down position. By placing the patient's arms at the sides with a support board (2 cm thickness) under the patient's back, the SBP could form the forced arching back to avoid high attenuation of arms in the cardiac segment by aligning the two humerus bones with the spinal column which was confirmed by the analysis of scout images and image noise. Our study demonstrated that a routine 100 kVp CTA setting could be maintained in the combined CTA with SBP.

No statistically significant difference was observed in the image noise of the left atrium between combined CTA, which employed a higher dose, and SBP combined CTA, suggesting that SBP can effectively mitigate image noise in the designated region. SBP‐combined CTA also exhibited no statistically significant difference in the CNR for coronary arteries compared with standard coronary CTA. The image noise variations between the left atrium and ascending aorta were similar for standard coronary CTA and SBP combined CTA, and significantly lower than those in combined CTA, suggesting that the SBP contributed to maintaining consistent image quality. We also observed the the image quality of craniocervical arteries in SBP combined CTA was superior to that of standard craniocervical CTA. The enhancement was attributed to the high HU resulting from the scanning protocol of SBP combined CTA, wherein coronary scan preceding craniocervical scan. As a result, the craniocervical arteries were scanned during the late arterial phase. This sequencing resulted in higher CT values for the craniocervical arteries, thereby improving image quality.^28^, ^29^

The SBP proposed in this study for scanning is applicable not only to combined CTA of coronary and craniocervical arteries, but can also be extended to other scans. For example, adopting SBP during chest or abdominal scans could improve image quality for patients with limited mobility or physical discomfort, especially for the region of most interest. Moreover, the proposed SBP may also benefit protocols that place dynamic monitoring scans at the chest level, such as craniocervical CTA. This facilitates more precise triggering of scan through the unobstructed view of the chest by aligning the humerus bones with the spinal column. SBP could also provide an approach to reduce the influence of arm position in PET/CT scanning which was mitigated by advanced algorithms currently.^30^, ^31^

In this study, certain limitations should be acknowledged. Firstly, the evaluation of image quality and radiation does not considered emerging deep learning‐based CT reconstruction methods. Secondly, this study was only performed on one vendor and one scanner model. Thirdly, this study did not include a variety of positions.

In conclusion, for one‐stop coronary and craniocervical CTA, our proposed support bridge position, as opposed to the naturally arm‐down position, presented advantages in image quality and dose efficiency. Our study could provide guidance for the clinical practice of one‐stop coronary and craniocervical CTA protocol in the future.

## AUTHOR CONTRIBUTIONS

**Heng Zhou**: Writing—original draft preparation and data curation. **Cheng Yan**: Methodology; visualization; and design of the work. **Min Ji**: Writing—reviewing and editing and validation. **Zhang Shi**: Supervision. **Chun Yang**: Software. **Mengsu Zeng**: Investigation and conceptualization.

## CONFLICT OF INTEREST STATEMENT

The authors declare no conflicts of interest.

## Supporting information



Supporting Information

Supporting Information

## Data Availability

Data generated or analyzed during the study are available from the corresponding author by request.
