# Supplementary material for: Supported bridge position in one‐stop coronary and craniocervical CT angiography: A randomized clinical trial
Source: J Appl Clin Med Phys. 2024 Nov 15;26(1):e14561. doi: 10.1002/acm2.14561 (PMC11713983; doi:10.1002/acm2.14561)
Supplement: Supplementary file 1 — Supporting Information [file ACM2-26-e14561-s002.docx]

**Supplement**

**Table S1** SNR of three groups

|  |  | Group1 | Group2 | Group3 | P value | $P_{1}$ | $P_{2}$ | $P_{3}$ |
| --- | --- | --- | --- | --- | --- | --- | --- | --- |
| SNR | AAO | 14.49±2.09 | 12.14±2.01 | 13.62±2.62 | <.001 | <.001 | .16 | .005 |
|  | DAO | 13.41±1.73 | 10.54±1.77 | 11.62±2.12 | <.001 | <.001 | .02 | <.001 |
|  | LM | 14.28±1.93 | 11.59±2.08 | 13.52±2.44 | <.001 | <.001 | .22 | <.001 |
|  | LAD | 14.22±2.13 | 11.76±2.65 | 13.16±2.04 | <.001 | <.001 | .07 | .01 |
|  | LCX | 14.28±1.93 | 11.59±2.08 | 13.52±2.44 | <.001 | <.001 | .22 | <.001 |
|  | RCA | 14.29±2.12 | 12.09±2.16 | 13.37±2.49 | <.001 | <.001 | .13 | .02 |
|  | dRCA | 13.88±2.73 | 11.86±2.64 | 12.85±2.59 | <.001 | .001 | .16 | .18 |
|  | AOA | 27.21(23.87-31.76) | 30.01(25.21-37.57) | 36.1(29.49-44.57) | <.001 | .09 | <.001 | .03 |
|  | CCA | 38.92±6.93 | 36.74±12.42 | 52.74±17.66 | <.001 | .83 | <.001 | .002 |
|  | V1 | 36.87(33.30-41.34) | 35.17(24.67-41.96) | 46.22(35.05-63.16) | <.001 | .83 | <.001 | .002 |
|  | $\mathrm{CCA}_{\mathrm{bif}}$ | 54.86(48.59-60.07) | 59.89(47.74-65.8) | 77.78(62.23-95.76) | <.001 | >.99 | <.001 | <.001 |
|  | V2 | 54.47(48.37-59.11) | 60.48(48.41-65.85) | 74.66(54.84-88.51) | <.001 | .44 | <.001 | <.001 |
|  | ICA | 29.12±5.02 | 30.53±6.95 | 39.1±8 | <.001 | .83 | <.001 | <.001 |
|  | BA | 26.92±4.86 | 29.02±5.89 | 35.71±7.56 | <.001 | .27 | <.001 | <.001 |
|  | M1 | 28.42±4.72 | 31.39±7.62 | 36.92±8.27 | <.001 | .07 | <.001 | .01 |

The significance between Group 1 and Group 2 is represented by $P_{1}$, the significance between Group 1 and Group 3 is represented by $P_{2}$, and the significance between Group 2 and Group 3 is represented by $P_{3}$. All pairwise comparisons underwent Bonferroni correction.

AAO = ascending aorta, DAO = descending aorta, LM = left main, LAD = left anterior descending, LCX = left circumflex, RCA = right coronary artery, dRCA = distal right coronary artery, AOA = ascending aorta, CCA = original segment of the common carotid artery, V1 = preforaminal segment of the vertebral artery, $\mathrm{CCA}_{\mathrm{bif}}$ = common carotid artery at the bifurcation, V2 = foraminal segment of the vertebral artery, ICA = internal carotid artery, BA = basal artery, M1 = the M1 segment of the middle cerebral artery.

**Table S2** Subjective evaluation of image quality

|  | Group1 | Group2 | Group3 | P value |
| --- | --- | --- | --- | --- |
| pRCA | 3.45±0.44 | 3.61±0.46 | 3.60±0.49 | .11 |
| mRCA | 3.31±0.47 | 3.36±0.47 | 3.43±0.48 | .4 |
| dRCA | 3.18±0.46 | 3.22±0.54 | 3.30±0.59 | .1 |
| R-PDA | 3.35±0.51 | 3.40±0.77 | 3.31±0.62 | .36 |
| LM | 3.60±0.42 | 3.69±0.46 | 3.67±0.45 | .34 |
| pLAD | 3.62±0.46 | 3.67±0.46 | 3.55±0.46 | .37 |
| mLAD | 3.34±0.45 | 3.43±0.46 | 3.51±0.39 | .12 |
| dLAD | 3.20±0.39 | 3.29±0.40 | 3.28±0.44 | .49 |
| D1 | 3.19±0.42 | 3.28±0.45 | 3.20±0.46 | .43 |
| D2 | 3.16±0.38 | 3.23±0.46 | 3.17±0.63 | .88 |
| pLCX | 3.61±0.44 | 3.62±0.46 | 3.57±0.48 | .91 |
| OM1 | 3.00±0.30 | 3.14±0.55 | 2.96±0.62 | .15 |
| dLCX | 3.18±0.43 | 3.15±0.46 | 2.95±0.71 | .26 |
| OM2 | 3.01±0.47 | 3.03±0.47 | 2.90±0.50 | .4 |
| L-PDA | 2.97±0.53 | 3.10±0.65 | 2.94±0.42 | .64 |
| RI | 3.29±0.66 | 3.36±0.53 | 3.29±0.65 | .94 |
| CCA | 3.94±0.19 | 3.93±0.22 | 3.99±0.07 | .24 |
| V2 | 3.96±0.20 | 4.00±0.00 | 3.94±0.20 | .05 |
| ICA | 3.92±0.25 | 3.92±0.27 | 3.83±0.33 | .08 |
| BA | 3.87±0.28 | 3.93±0.24 | 3.85±0.31 | .16 |
| M1 | 3.91±0.26 | 3.99±0.07 | 3.92±0.26 | .13 |

pRCA = proximal right coronary artery, mRCA = mid right coronary artery, dRCA = distal right coronary artery, R-PDA = posterior descending artery from RCA, LM = left main, pLAD = proximal left anterior descending artery, mLAD = mid left anterior descending artery, dLAD = distal left anterior descending artery, D1 = first diagonal branch, D2 = second diagonal branch, pLCX =proximal left circumflex, OM1 = first obtuse marginal branch, dLCX =distal left circumflex, OM2 = second obtuse marginal branch, L-PDA = posterior descending artery from LCX, RI = ramus intermedius, CCA = common carotid artery, V2 = foraminal segments of the vertebral artery, ICA = internal carotid artery, BA = basal artery, M1= the M1 segment of the middle cerebral artery.
