# Supplementary material for: Supported bridge position in one‐stop coronary and craniocervical CT angiography: A randomized clinical trial
Source: J Appl Clin Med Phys. 2024 Nov 15;26(1):e14561. doi: 10.1002/acm2.14561 (PMC11713983; doi:10.1002/acm2.14561)
Supplement: Supplementary file 2 — Supporting Information [file ACM2-26-e14561-s001.docx]

**Supplement**


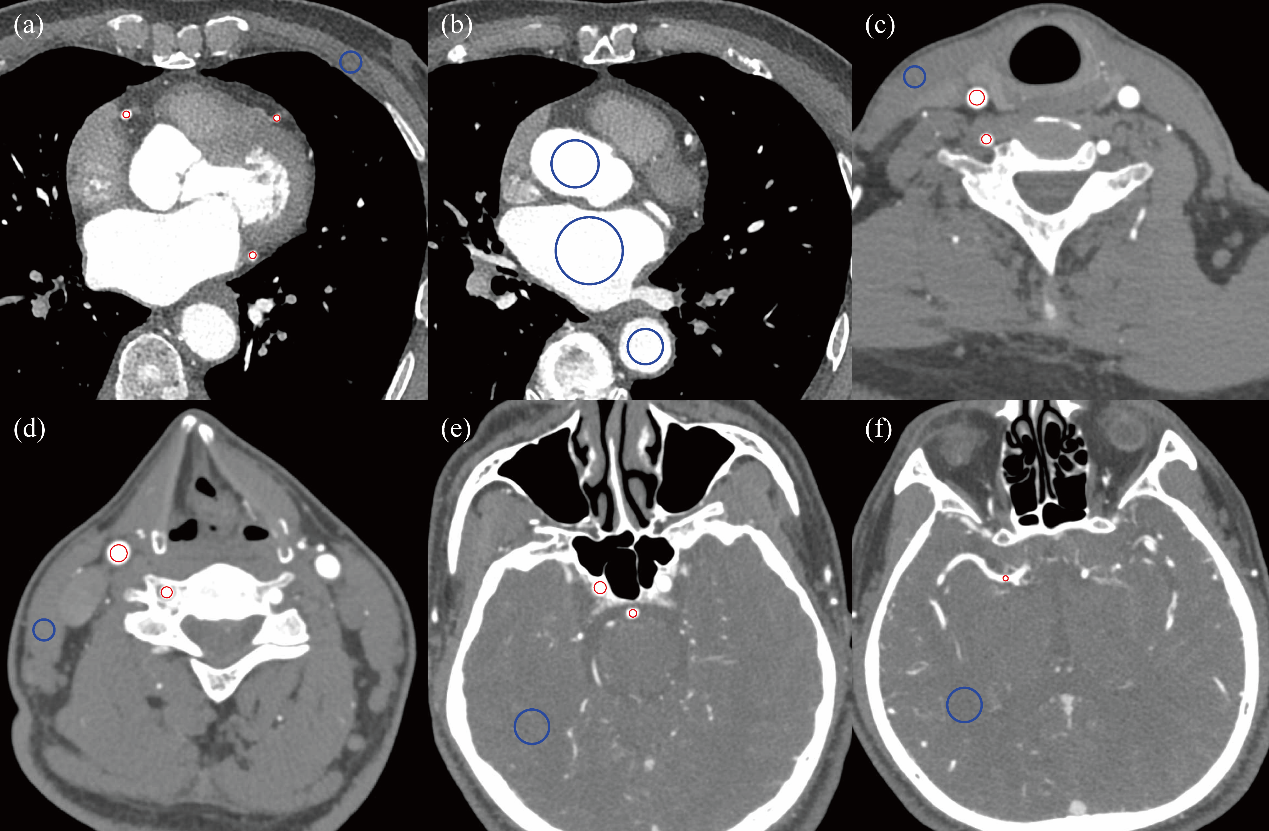


**Figure S1:** Regions of interest delineated for measuring the objective image quality. Red circles and bule circles respectively indicate regions of interests on vessels and muscles. a. for coronary arteries; b. for image noises in cardiac segment; c. at the base of the neck level; d. at the bifurcation level of the carotid artery; e at the brainstem level; f. at the cerebral level.

**
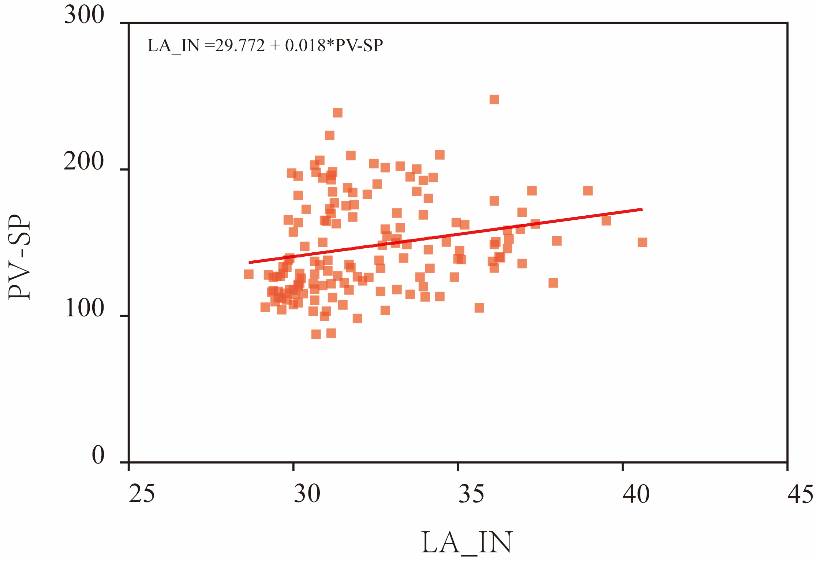
**

**Figure S2:** LA_IN in coronary arteries was proportional to the PV-SP.

PV-SP = project value in sagittal position, LA-IN = the image noise in the left atrium.
